# The *Toll*→NFκB Signaling Pathway Mediates the Neuropathological Effects of the Human Alzheimer's Aβ42 Polypeptide in *Drosophila*

**DOI:** 10.1371/journal.pone.0003966

**Published:** 2008-12-17

**Authors:** Lihua Tan, Paul Schedl, Ho-Juhn Song, Dan Garza, Mary Konsolaki

**Affiliations:** 1 Department of Molecular Biology, Princeton University, Princeton, New Jersey, United States of America; 2 Novartis Institutes for Biomedical Research, Cambridge, Massachusetts, United States of America; 3 Department of Genetics, Rutgers, The State University of New Jersey, Piscataway, New Jersey, United States of America; Massachusetts General Hospital and Harvard Medical School, United States of America

## Abstract

Alzheimer's (AD) is a progressive neurodegenerative disease that afflicts a significant fraction of older individuals. Although a proteolytic product of the Amyloid precursor protein, the Αβ42 polypeptide, has been directly implicated in the disease, the genes and biological pathways that are deployed during the process of Αβ42 induced neurodegeneration are not well understood and remain controversial. To identify genes and pathways that mediated Αβ42 induced neurodegeneration we took advantage of a *Drosophila* model for AD disease in which ectopically expressed human Αβ42 polypeptide induces cell death and tissue degeneration in the compound eye. One of the genes identified in our genetic screen is *Toll* (*Tl*). It encodes the receptor for the highly conserved *Tl→NFkB* innate immunity/inflammatory pathway and is a fly homolog of the mammalian Interleukin-1 (Ilk-1) receptor. We found that *Tl* loss-of-function mutations dominantly suppress the neuropathological effects of the Αβ42 polypeptide while gain-of-function mutations that increase receptor activity dominantly enhance them. Furthermore, we present evidence demonstrating that *Tl* and key downstream components of the innate immunity/inflammatory pathway play a central role in mediating the neuropathological activities of Αβ42. We show that the deleterious effects of Αβ42 can be suppressed by genetic manipulations of the *Tl→NFkB* pathway that downregulate signal transduction. Conversely, manipulations that upregulate signal transduction exacerbate the deleterious effects of Aβ42. Since postmortem studies have shown that the *Ilk-1→NFkB* innate immunity pathway is substantially upregulated in the brains of AD patients, the demonstration that the *Tl→NFkB* signaling actively promotes the process of Αβ42 induced cell death and tissue degeneration in flies points to possible therapeutic targets and strategies.

## Introduction

Although the neurodegenerative Alzheimer's disease (AD) is usually associated with the aging process, mutations in a number of different genes have been correlated with a familial, early onset of the dementia [1]–[5]. The first AD-related mutations identified were in the amyloid β precursor protein (APP), a type I integral membrane protein [3]–[6]. The APP protein is a precursor for a 42 residue proteolytic cleavage product, Aβ42, that accumulates in large aggregates or plaques in the brains of Alzheimer's patients. Mutations in the APP protein which are associated with early onset of AD increase the production of Aβ42 polypeptide or its tendency to form aggregates [7]–[10]. The connection between APP and, in particular, the Aβ42 polypeptide and AD was further strengthened by the finding that mutations in presenlin, a protease involved in the processing of the APP protein, are also associated with familial early onset AD [11]–[14]. These mutations are thought to increase the amount of Aβ42 polypeptide produced relative to another non-toxic APP cleavage product, the Aβ40 polypeptide.

While these observations have implicated Aβ42 in AD, how this polypeptide induces neurodegeneration remains uncertain. The brains of individuals with AD are characterized by large plaques of aggregated Aβ42 protein, neurofibrillary tangles composed of hyperphosphorylated Tau protein, and the loss of a significant fraction of the neurons in the hippocampus, prefrontal and entorhinal cortex [15]–[19]. Initially it was thought that the aggregated Aβ42 containing amyloid plaques induced the neurofibrillary tangles and neurodegeneration. However, the connection between large amyloid plaques and AD is unlikely to be direct as the correlation between the extent of plaque formation and either the severity of dementia or the loss of neurons is poor. Instead, recent studies have suggested that the large plaques may actually be relatively inert and that smaller oligomers of the Aβ42 polypeptide correspond to the primary neurotoxic agent for AD [20]–[22].

If the Aβ42 polypeptide is a causative agent for AD, it is important to understand what biological pathways are targeted by the polypeptide and how these pathways are deployed to produce the neuropathological phenotypes associated with the disease. With this aim in mind several laboratories have independently developed Alzheimer's models in the fruit fly *Drosophila* [23]–[26]. Ectopic expression of human Aβ42 polypeptide in the CNS of the fly was found to cause a range of phenotypes including a progressive decline in locomotor function, age-dependent learning defects, progressive neurodegeneration and loss of neurons and a significant reduction in lifespan. The expression of this protein was accompanied by the formation of diffuse amyloid deposits that seemed to be composed primarily of Aβ42 oligomers in younger animals, while in older animals small AD-like plaques. Further supporting the potential value of this *Drosophila* model for human AD, the deleterious effects of the Aβ polypeptide are specific to the Aβ42 isoform. Thus, unlike Aβ42, ectopic expression of the human Aβ40 polypeptide did not disrupt locomotor function or alter the lifespan, nor did it appear to induce neurodegeneration [26].

The neuropathological effects of the human Aβ42 polypeptide are not limited to the CNS. When Aβ42 is expressed in the eye it causes cell death and tissue degeneration [25], [26]. This disrupts the stereotypic and repeating morphology of the lens and gives a readily visible rough eye phenotype that worsens with age. Within a single transgenic line the severity of the rough eye phenotype is quite similar amongst flies of the same age; however, the phenotype depends upon the level of expression of the transgene insert and some inserts have only mild effects on eye development while others have moderate or even severe effects. Since the rough eye phenotype is sensitive to changes in Aβ42 activity it is well suited for identifying genes that modify the pathological effects of the Aβ42 polypeptide. This phenotypic assay has been used to screen a collection of nearly 2,000 EP transposon strains for insertions that alter the Aβ42-induced rough eye phenotype. The EP transposon has a GAL4 activated promoter and, depending upon the site and orientation of the insertion, it will upregulate gene activity, have no effect, or downregulate gene activity. Altogether 23 lines that modified the rough eye phenotype were recovered in the EP screen. The interacting EP insertions were found to be located in genes involved in secretion, cholesterol homeostatis, the innate immune pathway and chromatin organization [27].

As a complementary approach for elucidating the genes and pathways that mediate the neurodegenerative effects of the Aβ42 polypeptide, we have screened for second site loss-of-function mutations that dominantly enhance or suppress the degeneration of the eye induced by the Aβ42 polypeptide. To identify genes important for Aβ42 neuropathology we first screened the Bloomington stock center 2^nd^ and 3^rd^ chromosome deficiency kit for interacting deletions. We then pinpointed the locus responsible for modifying the rough eye phenotype by testing mutations in genes that are included in the deficiency. One of the suppressing deficiencies uncovered the *Drosophila Toll* (*Tl*) gene. *Toll* encodes a transmembrane receptor which has a leucine rich extracellular domain and intracellular signaling domain that is closely related to the mammalian Interleukin-1 receptor [28]–[31]. The *Tl* gene was first identified in maternal effect screens because of its role in establishing the dorsal-ventral (D-V) polarity axis of the embryo [32], [33]. In D-V polarity, binding of Spatzle ligand to the Toll extracellular domain activates a cytoplasmic signaling cascade [34]. This cascade promotes the nuclear translocation of two NFκB-like transcription factors, Dorsal and Dif, which function to specify ventral cell fate in blastoderm stage embryo [35]–[36]. Subsequent work demonstrated that in addition to its role in embryonic polarity, the *Tl* receptor→NFκB signal transduction pathway is deployed, just as it is in mammals, in the innate immunity and inflammatory pathways of the fly [28]–[31], [38]–[40].

In the studies reported here we show that the *Tl*→NFκB innate immunity-inflammatory pathway plays a central role in orchestrating the neuropathological activities of the human Aβ42 polypeptide in flies. Our findings support emerging models for AD and other forms of neurodegenerative diseases in humans in which the inflammatory response acts as a critical catalyst in promoting the process of neurodegeneration.

## Results

### Reducing Tl activity ameliorates the neuropathological effects of the Aβ42 polypeptide on eye development

**Figure 1:**
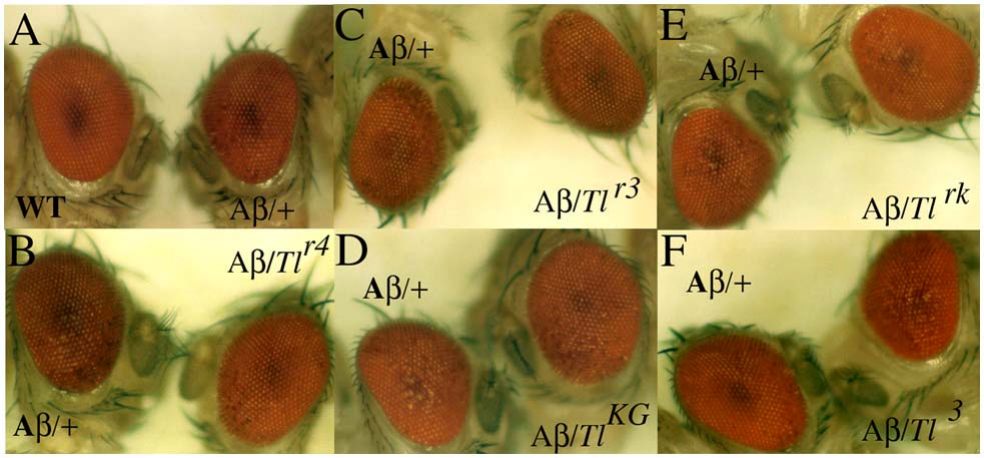
Aβ42 induced rough eye phenotype is sensitive to *Toll* activity. WT: Wild type flies. Aβ: pGMR-Aβ42 transgenic flies. In the experiments shown in this figure, the flies are hemizygous for the pGMR-Aβ42 transgene. Panel A: WT: eye of a wild type fly. Aβ/+: eye of a wild type fly hemizygous for the pGMR-Aβ42 transgene. Panels B through F show sibling pGMR-Aβ42 transgene flies that are either wild type *Tl* or heterozygous for the indicated *Tl* allele. Panel B: Aβ/+: hemizygous for the Aβ transgene; Aβ/*Tl^r4^*: heterozygous for the LOF allele *Tl^r4^*. Panel C: Aβ/+: hemizygous for the Aβ transgene; Aβ/*Tl^r3^*: heterozygous for the LOF allele *Tl^r4^*. Panel D: Aβ/+: hemizygous for the Aβ transgene; Aβ/*Tl^KG^*: heterozygous for the LOF allele *Tl^ KG03609^*. Panel E: Aβ/+: hemizygous for the Aβ transgene; Aβ/*Tl^rk^*: heterozygous for the LOF allele *Tl^ rK343^*. Panel F: Aβ/+: hemizygous for the Aβ transgene; Aβ/*Tl^3^*: heterozygous for the GOF allele *Tl^ r3^*.

Ectopic expression of Aβ42 in the developing eye using the Glass Multimer Reporter, pGMR, induces a rough eye phenotype [25], [26]. This phenotype can be used to screen for interacting genes that mediate the neurodegenerative effects of the Aβ42 polypetide on the *Drosophila* eye. Since we wished to identify mutations in genes that can either ameliorate or potentiate the effects of the Aβ42 polypeptide, we selected a pGMR:Aβ42 insertion that has a moderate rough eye phenotype (Fig. 1A). We used this insert to screen the Fly Center collection of 2^nd^ and 3^rd^ chromosome deficiencies (which is thought to account for ∼60% of fly genes) for deletions that dominantly (Df/+) suppress or enhance the Aβ42 eye phenotype. Of the 130 deficiencies tested, we identified 14 strong or moderate suppressors and 9 strong or moderate enhancers.

One of the strong suppressors was *Df(3)T1-P* which uncovers the cytological interval 97A-98A on the right arm of the 3^rd^ chromosome. To further narrow down the chromosomal DNA segment that contains the interacting gene(s) we tested five deficiencies that overlap *Df(3)T1-P*–*Df (3)Tl-l* (97B-97E), *Df(3R)T1-X* (97B-97D1,2), D*f(3R)ED6235* (97B9-97D1,2), *Df(3R)ro80b* (97D1-D13) and *Df(3R)ME61* (96F1,2-97C5). All but *Df(3R)ME61* ameliorated the rough eye phenotype and taken together these findings map the suppressor to the 97D interval.

To identify the suppressor we next tested mutations in genes that are known to be located in this chromosomal interval. One of the genes that is included in the 97D and is deleted in all of the suppressing deficiencies is *Tl*. This gene was the most plausible candidate as one of the EP transposons uncovered in the previous screen is inserted into the *Tl* gene [27]. *Tl* encodes a highly conserved type I transmembrane receptor that shares extensive homology with vertebrate receptors for the cytokines interleukin-1 (IL-1) and interleukin 18 (IL-18) [29], [41], [42]. In flies and vertebrates the Toll-like receptors are components of the innate immune response/inflammatory pathway that control the nuclear localization of transcription factors in the NFκB family [41]–[45].

The first *Tl* mutation we tested, *Tl^r4^*, is an ethylmethanesulfonate (EMS) induced allele. It is a hypomorphic mutation that has two amino acid substitutions in the extracellular domain of the receptor [46]. As shown in Fig. 1B and Table 1, *Tl^r4^* strongly suppresses the rough eye phenotype induced by ectopic Aβ42. While this result suggested that *Tl* corresponds to the interacting locus, we wanted to exclude possible effects of genetic background. Thus to confirm the identity of the suppressor we tested three other *Tl* loss-of-function alleles. The first is a temperature sensitive EMS induced allele, *Tl^r3^*. The second and third are the transposon induced mutations, *Tl^KG03609^* and *Tl^rK343^*. The former is inserted into the 5′ UTR, while the latter is inserted into a large intron. Like the two EMS alleles, these insertions are hypomorphic. As shown in Fig. 1C–E (and Table 1), all three of these mutations also suppress the Aβ42 induced rough eye phenotype. *Tl^r3^* and *Tl^KG03609^* are weak suppressors, while suppression by *Tl^rK343^* is almost as strong as that observed for *Tl^r4^*. The differences in the extent of suppression between these hypomorphic *Tl* alleles are likely to be due to a combination of both the strength of the mutation and differences in their genetic backgrounds.

**Table 1:** *Tl* amd *dl* mediate the Aβ42 induced rough eye phenotype.

Aβ42/+	++++
Aβ42/*Tl^r4^*	+
Aβ42/*Tl^r3^*	+++
Aβ42/*Tl^KG03609^*	+++
Aβ42/*Tl^rk343^*	++
Aβ42/*Tl^3^*	++++++
Aβ42/*dl^1^*	++/+++
Aβ42/*dl^4^*	++
Aβ42/*dl^8^*	++/+++
Aβ42/*hsp83:dl*	++++++

The rough eye phenotype of pGMR-Aβ42 flies was assigned a value of ++++. According to this scoring system, strong, moderate and weak suppression corresponded to +, ++, and +++ respectively. Weak and moderate enhancement corresponded to +++++ and ++++++. All flies are hemizygous for the transgene and either wild type (+) or heterozygous for the indicated mutation. In the experiment with the *hsp83:dl* transgene the flies are hemizygous for this transgene.

### Excess Tl activity enhances the neuropathological effects of Aβ42

The finding that several independent loss-of-function mutations in *Tl* dominantly suppress the rough eye phenotype induced by ectopic Aβ42 is intriguing as connections between AD and canonical mammalian innate immunity/inflammatory pathways have been extensively documented in the literature [45]–[49]. However, while these studies clearly demonstrate that the inflammatory pathways are activated in the brains of AD patients, it is not clear from this correlation whether the deployment of these pathways actively promotes the process of neurodegeneration or whether they are functioning instead to retard the progression of the disease [47]–[52]. Thus, it would be important to determine whether there is actually a causal relationship between the activation of the fly *Tl*→NFκB innate immunity/inflammatory pathway and the disruptive effects of Aβ42 on eye development.

If the fly *Tl*→NFκB signaling pathway functions to actively promote the neuropathological effects of Aβ42, then excess *Tl* activity would be expected to exacerbate the Aβ42 induced rough eye phenotype. Consistent with this prediction, the transposon insertion in *Tl* identified in the EP screen strongly enhances the rough eye phenotype. Since the transposon upregultates *Tl* mRNA by nearly 5 fold, this would be expected to substantially increase the amount of Tl protein. On the other hand, while elevated levels of the receptor should potentiate signal transduction, we cannot exclude the possibility that Aβ42 or some side product induces neurodegeneration by some type of interaction with Tl that does not involve an activation of the receptor. To test this possibility we combined the pGMR-Aβ42 transgene with a gain-of-function *Tl* allele, *Tl^3^*. This allele has an amino acid substitution in the extracellular domain that leads to constitutive signaling by the Tl receptor independent of ligand [31], [45]. As shown in Fig. 1F (and Table 1) excess Tl signaling enhances the neuropathological effects of the Aβ42 polypeptide.

### Neuropathological effects of Aβ42 are mediated by the Tl signaling pathway

The effects of reducing and increasing *Tl* activity would be consistent with the idea that the fly *Tl*→NFκB signaling pathway plays an instrumental role in promoting Aβ42 induced cell death and tissue degeneration in the eye. This hypothesis makes two strong predictions. The first is that the severity of the Aβ42 rough eye phenotype will be sensitive to the activity of other components of the *Tl*→NFκB signaling pathway. The second is that genetic manipulations which lead to an upregulation of *Tl*→NFκB signal transduction will tend to exacerbate the rough eye phenotype, while manipulations that downregulate signal transduction will tend ameliorate the phenotype.

**Figure 2:**
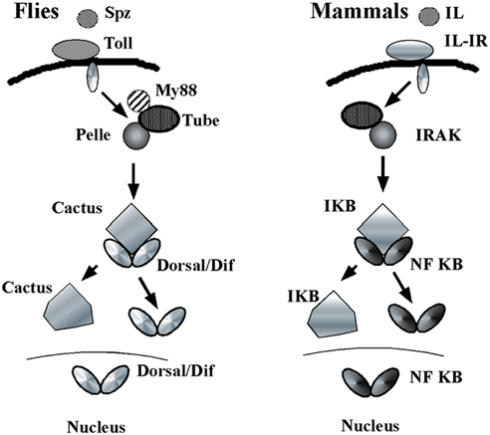
The *Drosophila* Toll→Dorsal/Dif and mammalian Interleukin Receptor→NFκB innate immunity pathways. Diagram of the *Tl*→NFκB signaling pathways in *Drosophila* and mammals. See text for details.

As shown in Fig. 2, a circulating peptide, Spatzle, which is activated by proteolysis binds to the Tl receptor and turns on the cytoplasmic signaling cascade [28]–[31], [33]. Signal transduction depends upon an adaptor protein Tube (Tub) and the IRAK-like kinase Pelle (Pll). The target for the Pll kinase is the fly IκB homolog Cactus (Cact). In the absence of signaling, the Cact protein binds to the NFκB family transcription factors Dl and Dif and retains them in the cytoplasm. Phosphorylation of Cact by Pll is thought to target Cact for degradation, and this releases Dl and Dif for nuclear translocation and regulation of their target genes.

***--dl:*** The function of the *Tl* signal transduction cascade in the innate immune response is to promote the nuclear translocation of the Dl and Dif transcription factors [28]–[31]. These two NFκB family members are thought to exist as Dl:Dl and Dif:Dif homodimers and Dl:Dif heterodimers. Studies by Han and Ip [53] have suggested that the homo and heterodimers regulate different sets of genes and consequently either one or both of these NFκB transcription factors could be the relevant target for the *Tl* pathway in Aβ42 induced neuropathology.

**Figure 3:**
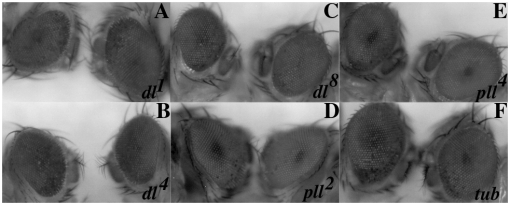
Components of the *Toll-NF*κ*B* signaling pathway modulate Aβ42 polypetide induced neurodegeneration. Panels A–F: In the experiments shown in this figure all flies are siblings that are hemizygous for the pGMR-Aβ42 transgene. The fly on the left side of each panel is a wild type sib, while the fly on the right side of each panel is a sib that is heterozygous for the indicated mutation in the *Toll-NF*κ*B* signaling pathway mutant. Panel A: *dl^1^*. Panel B: *dl^4^*. Panel C: *dl^8^*. Panel D: *pll^2^*. Panel E: *pll^7^*. Panel F: *tub*.

If the *Tl* dependent nuclear translocation of Dl promotes the neuropathological effects of Aβ42 then reducing the activity of this transcription factor would be expected to suppress the rough eye phenotype. We tested three different *dl* alleles *dl^1^*, *dl^4^* and *dl^8^* for genetic interactions with the pGMR-Aβ42 transgene as heterozygotes. As shown in Fig. 3 and Table 1, *dl^4^* is a moderate suppressor, while the two other *dl* alleles are moderate to weak suppressors.

**Figure 4:**
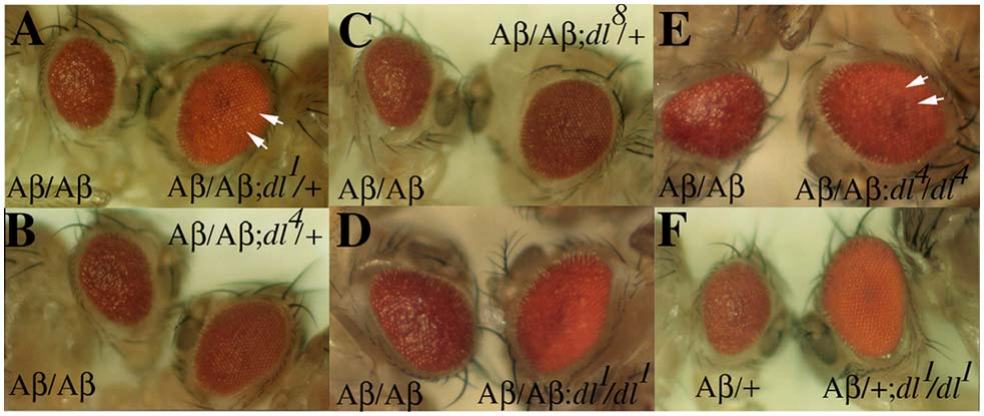
Dorsal mediates Aβ42 dependent neurodegeneration of the eye. Panels A–E: In the experiments shown in this figure all flies are siblings that are homozygous for the pGMR-Aβ42 transgene. The fly on the left side of each panel is a wild type sib, while the fly on the right side of each panel is a sib that is either heterozygous or homozygous for the indicated *dl* mutation. Panel A: *dl^1^*/+. Panel B: *dl^4^*/+ Panel C: *dl^8^*/+ Panel D: *dl^1^*/*dl^1^*. Panel E: *dl^4^/dl^4^*. Panel F: In this experiment both flies are hemizygous for the pGMR-Aβ42 transgene. The fly on the left is wild type, while the fly on the right is homozygous *dl^ `^*. Arrows in panels A and E point to ommatidia with dead or dying cells.

The disruptions in eye development induced by the Aβ42 polypetide are dose sensitive and can be greatly enhanced when there are two copies of the pGMR-Aβ42 transgene. Unlike the hemizygotes (see Figs 1 & 3), the eyes of homozygous flies are smaller in size and instead of a regular repeating hexagonal array of ommatidia, the surviving ommatidia are highly disorganized and have a blistered and very glossy morphology (see Aβ/Aβ eyes in Fig. 4). We reasoned that if the *Tl→dl* pathway is a key player in Aβ42 induced neurodegeneration, then it would be possible to ameliorate even this much more severe disruption in eye development by reducing *dl* activity. This is the case. In fact, we found that reducing the dose of the *dl* gene from 2 to 1 wild type copy strongly suppresses the neuropathological effects of the Aβ42 polypeptide. This was observed not only for the moderate suppressor, *dl^4^*, but also for both of the weaker suppressors *dl^1^* and *dl^8^* (Fig. 4). In all three cases, the eye of the pGMR-Aβ42/pGMR-Aβ42; *dl/+* fly is nearly wild type in size. Moreover, instead of the very rough and glossy eye morphology evident in the Aβ42 homozygotes, the ommatidia of the *dl/+* flies are arranged in a considerably more regular and repeating hexagonal array across most of the eye. Suppression is not, however, complete and the eye morphology is not like wild type. For example, there are many speckles scattered across the *dl^1^/+* eye shown in Fig. 3A (see arrows) which correspond to ommatidia that have dead or dying cells. Similarly, though the ommatidia in the *dl^−^/+* flies shown in Fig. 3B & C have a much more regular organization than their *dl^+^* sibs, the surface of the eye still appears rough and glossy.

To further confirm that the *T→dl* pathway actively promotes the deleterious effects of the Aβ42 polypeptide, we generated pGMR-Aβ42/pGMR-Aβ42 trangenic flies that are also homozygous for the *dl^1^*, *dl^4^*, or *dl^8^* mutations. The severe disruptions in eye development induced by high levels of Aβ42 are strongly suppressed in flies that lack *dl* activity. This is shown for *dl^1^* and *dl^4^* in Fig. 4D and E. As can be seen in these panels, the eyes of the homozygous *dl* mutants show little evidence of the residual rough or glossy morphology that is seen in transgenic animals that still have a single wild copy of the *dl* gene. On the other hand, the eyes are not quite wild type and there are still some ommatidia that have a few dead or dying cells (see arrows). As might be expected, essentially complete suppression of the rough eye phenotype is observed in homogyzous *dl* mutants when there is only a single copy of the pGMR-Aβ42 transgene. As illustrated in Fig. 4F for the *dl^1^* allele, the eyes of hemizygous transgenic flies that are homozygous mutant for *dl* are essentially indistinguishable from wild type.

If reducing *dl* activity suppresses the neuropathological effects of the Aβ42 polypeptide, then increasing *dl* activity might be expect to enhance its effects. To test this possibility we introduced an *hsp83:dl* transgene into flies carrying the pGMR-Aβ42 transgene. As shown in Table 2, we found that ectopic *dl* enhanced the Aβ42 rough eye phenotype.

**Table 2:** Effect of mutations in the *Tl→ΝFκB* pathway on the Aβ42 induced rough eye phenotype.

Aβ42/*+*	++++
Aβ42/*dif^1^*	+++
Aβ42/*tub^2^*	++
Aβ42/*pll^2^*	++/+++
Aβ42/*pll^7^*	++
Aβ42/*tub pll*	+

The rough eye phenotype of pGMR-Aβ42 flies was assigned a value of ++++. According to this scoring system, strong, moderate and weak suppression corresponded to +, ++, and +++. Weak and moderate enhancement corresponded to +++++ and ++++++. All flies are hemizygous for the transgene and either wild type (+) or heterozygous for the indicated mutation.

***--dif:*** Since Dif:Dl heterodimers and Dif:Dif homodimers are also thought to mediate the *Tl*-dependent innate immune response pathway, but regulate different target genes than Dl:Dl homodimers, it was of interest to test whether reducing *dif* activity also suppressed the Aβ42 rough eye phenotype. Consistent with the idea that *dif* is also an important *Tl* target in Aβ42 induced neurodegeneration we found *dif^1^* is a weak suppressor (Table 1).

***--tub and pll:*** The *tub* and *pll* gene products function inside the cell and are required for signal transduction by the Tl receptor. We tested *tub^2^* which is thought to be amorphic allele. As shown in Fig. 2F (and Table 1), this mutation is a moderate suppressor of the Aβ42 rough eye phenotype. We also tested two *pll* alleles, *pll^2^* and *pll^7^*. The former is a weak to moderate suppressor, while the latter is a moderate suppressor (Fig 2D & E and Table 2). Thus, as would be predicted if the *Tl*→NFκB pathway functions to promote the neuropathological effects of the Aβ42 polypetide, reducing the activity of *tub* and *pll* suppresses the rough eye phenotype.

Since the reduction in *tub* and *pll* acitivty in these mutants is at most only 2-fold, it seemed possible that this would not downregulate *Tl* signaling sufficiently to have a strong effect on the Aβ42 rough phenotype. For this reason, we took advantage of a recombinant chromosome that carries mutations in both *tub* and *pll*. As shown in Table 2, when the pGMR-Aβ42 transgene is introduced into *tub pll/++* flies, the rough eye phenotype is strongly suppressed.

**--** ***spz:*** We tested three alleles of the *spz* ligand, *spz^2^*, *spz^3^*, and *spz^KG05402^*. These *spz* mutations had no apparent effect on the rough eye phenotype as heterozygotes (not shown). In this case the failure to detect a genetic interaction could indicate that some other ligand is required to activate the Tl receptor in this instance. There are several other *spz*-like genes in *Drosophila* that could potentially perform this function [31]. On the other hand, this assay may demand that a two-fold reduction in gene dose alter the activity of the pathway to an extent sufficient to modify the rough eye phenotype. Thus, it also is possible that *spz* is not haploinsufficient.

***relish and imd:*** Flies have a second innate immune response pathway that is dependent upon the Imd receptor rather than the Tl receptor. We tested 4 mutations in the *imd* receptor but did not observe any obvious alteration in the Aβ42 rough eye phenotype (not shown). The Imd pathway regulates the nuclear import of the third fly NFκB-like transcription factor Relish (Rel). We also tested four different *rel* alleles, and like *imd* there was no apparent alteration in the eye phenotype when *rel* activity was reduced. While these findings could indicate that the neuropathological effects of the Aβ42 polypeptide are independent of the *Imd* innate immunity pathway, it is also possible that *imd* and *rel* are not haploinsufficient in this assay.

### Aβ42 upregulates Cact expression in fly heads

**Figure 5:**
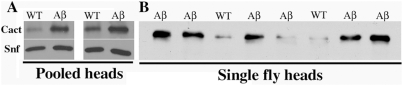
Cactus protein expression is induced by the Aβ42 polypeptide. Panel A. Extracts were prepared from the heads of 10 wild type flies (WT) or 10 flies that are homozygous for the pGMR-Aβ42 transgene (Aβ). After gel electrophoresis and blotting, the blots were probed with Cactus and Snf (Sans fille—the fly U1A/U2B″ snRNP protein) antibodies. The upper panel is Cactus, while the lower panel is the Snf loading control. Two independent experiments are shown. Quantitation indicates that the level of Cactus protein is about 3.5–5 fold higher in the heads of flies carrying the transgene. Panel B. Extracts were prepared from heads of single wild type or pGMR-Aβ42 transgenic flies. After gel electrophoresis and blotting the blots were probed with Cactus (and Snf: not shown) antibodies. Quantitation indicates that the level of Cactus protein in heads of individual transgenic flies was between 3–8 fold higher than in wild type.

In vertebrates one of the genes that is upregulated by the inflammatory response is IκB which is responsible for retaining NFκB in the cytoplasm [54]. This is also true in flies where activation of the *Tl* pathway following microbial infection is found to induce the expression of the fly IκB homolog Cact [55], [56]. Our results would argue that ectopic Aβ42 must also activate the *Tl*→NFκB innate immune response pathway. If this supposition is correct, then the expression of the fly IκB homolog Cact should be upregulated by Aβ42. To test this prediction we compared Cact protein accumulation in head extracts prepared from pooled samples of either 10 wild type flies or 10 transgenic flies that carry two copies of the pGMR-Aβ42 transgene. In wild type flies, only low levels of Cact protein are detected in head extracts. However, as evident from inspection of the two independent experiments shown in Fig. 5A, there is substantial increase in the levels of Cact protein in the heads of pGMR-Aβ42 flies compared to the wild type controls.

We wondered whether the induction of the inflammatory response by ectopic Aβ42 is stochastic and is upregulated in only subset of the flies, or whether most flies exhibit a similar reaction to the Aβ42 polypeptide. To investigate this question, we examined Cact expression in heads from individual wild type and Aβ42 transgene flies. The experiment in Fig. 5B shows Cact protein accumulation in heads from three wild flies and from six flies carrying two copies of pGMR-Aβ42 transgene. Cact protein levels are elevated compared to wild type in heads from five of the six transgenic flies. Altogether we examined Cact expression in heads from 22 wild type flies and 18 pGMR-Aβ42/pGMR-Aβ42 flies. In all of the wild type flies we observed only low levels of Cact protein comparable to the three examples shown in Fig. 5B. By contrast, Cact protein levels were elevated to an extent similar to that seen in the examples shown in Fig. 5B in 17 of the 18 transgenic flies. In other experiments we tested whether another marker for the induction of the inflammatory response, Dorsal, was also upregulated in the heads of pGMR-Aβ42/pGMR-Aβ42 flies. Elevated levels of Dl were observed in the heads of the 5 flies that were tested (data not shown).

While these findings indicate that the inflammatory response is consistently activated by ectopic Aβ42, it seemed possible that the efficiency or extent of induction would depend upon the dose of the Aβ42 protein. To address this question we examined Cact expression in the heads of transgenic flies that were hemizygous rather than homozygous for the pGMR-Aβ42 transgene. As might be expected if induction of the inflammatory response depends upon the dose of the Aβ42 protein, we found that only 13 of the 18 head extracts examined had higher levels of Cact protein than the wild type controls. We also noted that the level of Cact protein in the pGMR-Aβ42 hemizygotes did not appear to be as high as in the flies homozygous for the pGMR-Aβ42 transgene (not shown).

### Tl signaling pathway and life span

The age dependent neurodegeneration induced by ectopic expression of Aβ42 in the CNS using an *elav-GAL4* driver results in learning deficits, climbing disabilities and a shortened life span in adult flies [26]. Since the deleterious effects of ectopic Aβ42 in the eye are dependent upon activation of the *Tl* pathway, we wondered whether this was also true in the CNS. To address this question we asked whether modulating the activity of the *Tl* signaling pathway alters the life span defects observed in Aβ42 flies.

**Figure 6:**
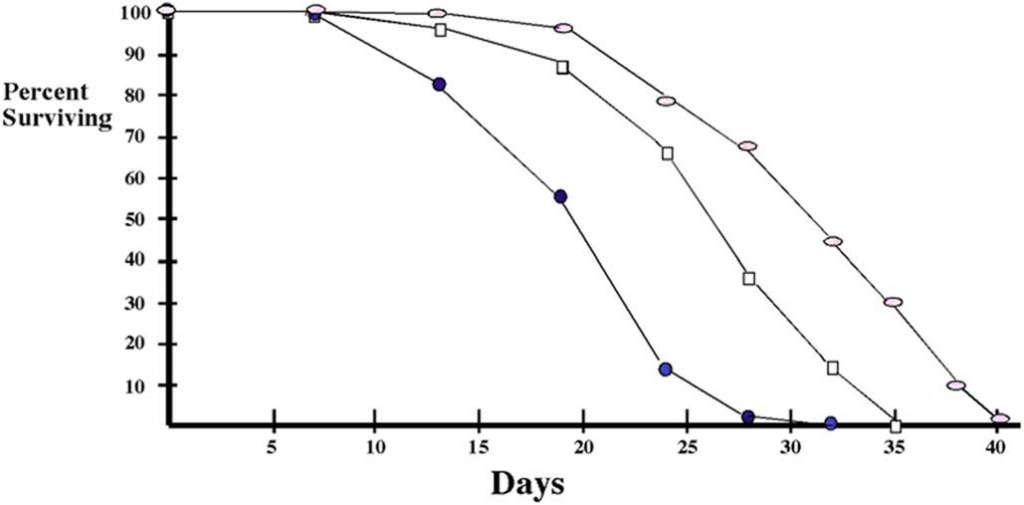
Reducing *Tl* activity partially ameliorates the reduction in lifespan induced by expressing the Aβ42 polypeptide in the CNS. Filled circles: UAS:Aβ42/*elav-GAL4* transgenic flies. Open boxes: UAS:Aβ42/*elav-GAL4* transgene flies heterozygous for *Tl^4^*. Ovals: *elav-GAL4* transgenic flies. Statistical tests using the Log Rank and Wilcoxon tests from the Lifetest Procedure both gave a Chi-Square of <0.0001 for the difference between the life span of UAS:Aβ42/*elav-GAL4* flies and of UAS:Aβ42/*elav-GAL4*; *Tl^4^/+* flies. Though small differences in life span (2–3 days or about 10%) were observed for two other *Tl* mutations and for mutations in *tub* and *pll*, the sample size was not large enough to be statistically significant.

As previously reported, flies carrying both the Aβ42 transgene and the *elav-GAL4* driver had a shorter average (50% survival at ∼19 days) lifespan than the *elva-GAL4* driver control (50% survival at ∼31 days) (see Fig. 6). We tested 3 different *Tl* loss of function alleles, *Tl^r4^*, *Tl^r3^* and *Tl^KG03609^*. In the eye assay *Tl^r4^* was a strong suppressor, while *Tl^r3^* and *Tl^KG03609^* were both weak suppressors. As shown in Fig. 6, we found that the life span of the *UAS-Aβ42*/*elav-GAL4* flies was extended by the *Tl^r4^* mutation (50% survival at 26 days). On the other hand, little if any effect on life span was observed for the two other *Tl* alleles, *Tl^r3^* and *Tl^KG03609^*. In both cases, the life span was extended by only a few days (or about a 10% increase). We also tested whether mutations in either *tub* or *pll* dominantly suppressed the Aβ42 induced life span defects. As was the case for the two weaker *Tl* alleles, *tub* and *pll* mutations had little effect, extending the average life span (50% surviving) by at most a 2–3 days.

## Discussion

While it has been known for more than two decades that there is a global upregulation of the inflammatory and innate immunity pathways in the brains of AD patients, it has not been clear what role, if any, the inflammatory response plays in AD neurodegeneration [46]–[51]. Over the years a number of findings have raised the possibility that the inflammatory response plays an active role in promoting neurodegeneration. One of the first indications that it could contribute to disease progression came from epidemiological studies indicating that prolonged treatment with anti-inflammatory drugs is correlated with a reduced risk of developing AD [57]–[59]. Experiments using either mammalian tissue culture cell or whole organism AD models have also pointed to a connection between Aβ induced neurodegeneration and activation of the inflammatory response [59]–[62]. These studies have shown that expression of the cytokines IL-1, IL-6 and TNF-α are upregulated by the human Aβ peptide and that induction of these cytokines both by Aβ and by other mechanisms can have deleterious effects on neuronal cell viability and cognition. Moreover, downregulation of the innate immune response pathway in a mouse AD model by treatment with anti-inflammatory drugs or disruption of the tumor necrosis factor death receptor gene was found to ameliorate the effects of ectopic Aβ [59], [62]–[4]. On the other hand, not all findings have been consistent with the notion that the inflammatory response/innate immunity pathways promote neurodegeneration. In fact, a number of reports have suggested instead that these pathways play a beneficial rather than deleterious role [48], [49], [52]. Thus, it is not clear at this point whether inflammation is a causative agent in the process of neurodegeneration, is an essentially benign response to AD, or is actually beneficial and acts to retard the progression of the disease [52].

The findings reported here strongly support the hypothesis that the *Tl*→NFκB innate immunity pathway plays a critical role in mediating the neuropathological effects of the human Aβ42 polypeptide. We identified this pathway in an unbiased genetic screen for mutations that either promote or suppress the neurpathological effects of the Aβ42 polypeptide on the *Drosophila* eye. One of the suppressors recovered in our genetic screen was the *Tl* receptor, which is a key component of the fly innate immunity pathway.

Several lines of evidence demonstrate a direct connection between the neuropathological effects of Aβ42 and the activation of the *Tl*→NFκB signaling cascade. First, the Aβ42 polypeptide induces the accumulation of one of the well known downstream transcriptional targets of the *Tl*→NFκB pathway, the fly IκB homolog *cact*. We also found that the levels of the fly NFκB protein Dl are upregulated in pGMR-Aβ42 transgenic flies as well. Thus, the response of flies to the neurotoxic effects of the Aβ42 polypeptide appears to mimic the upregulation of the inflammatory pathways evident in both AD patients and in mouse AD models [47]–[49], [59]–[63]. Moreover, the extent of *cact* induction depends upon the dose of the Aβ42 polypeptide. In flies carrying two copies of the pGMR-Aβ42 transgene *cact* accumulation is substantially elevated in virtually every fly. By contrast, in flies carrying only a single pGMR-Aβ42 transgene there is typically a smaller increase in the level of the Cact protein, and in a subset of the transgenic flies little change in Cact accumulation is evident.

Second, as would be predicted if activation of the inflammatory response mediates the neuropathological effects of the Aβ42 polypeptide, we found that loss-of-function mutations in *Tl* dominantly suppress neurodegeneration of the eye induced by ectopic Aβ42. Conversely, a *Tl* gain-of-function allele that signals constitutively independent of ligand exacerbates the degenerative effects of Aβ42. In this context, it is interesting to note that *Tl* was identified in the screen of EP insertions [27]. The *Tl* insertion was found to be a strong enhancer of Aβ42 induced degeneration of the eye and it substantially upregulates the expression of *Tl* mRNA. Thus, it is possible to enhance the sensitivity of the fly to the pathological effects of Aβ42 not only by increasing the activity of the Tl receptor, but also by increasing the amount of the receptor.

Third, Aβ42 induced neurodegeneration is mediated by the downstream target for the *Tl*→NFκB signaling cascade, the *dl* transcription factor. This is most clearly demonstrated in pGMR-Aβ42 flies that are homozygous for *dl* mutations. When there is only a single copy of the transgene, suppression of the Aβ42 rough eye phenotype by the *dl* mutation is nearly complete and the eyes resemble wild type. Degeneration of the eye is dependent on the dose of the Aβ42 polypeptide and is much more severe when there are two copies of the pGMR-Aβ42 transgene. However, even in this case strong suppression is observed when the transgenic flies are homozygous for a *dl* loss of function mutation. Moreover, this is not due to genetic background as three independent *dl* alleles substantially reduce the much more extreme neuropathological effects produced by two copies of the pGMR-Aβ42 transgene. The effects of *dl* are not limited to homozygous mutant flies; we also found that the pGMR-Aβ42 induced disruptions in eye development are suppressed when the flies are heterozygous for a *dl* mutation. In this case, suppression is less complete than that observed in flies that are homozygous for the same *dl* mutation.

Fourth, like *Tl* and *dl*, mutations in three other components of the *Tl*→NFκB signaling cascade, *tub*, *pll* and the partner of the *dl* transcription factor, *dif* also dominantly suppress the degenerative effects of Aβ42 on eye development. In addition, one of the chromatin modifiers recovered in the EP screen, Dsp1, functions as a Dl dependent co-repressor [27], [65]. It should be noted that not all genes in the *Tl*→NFκB signaling cascade show dominant genetic interactions with pGMR-Aβ42. We tested three different mutations in the *Tl* ligand *spz*; however, no alterations in the rough eye phenotype were observed. Similarly no effects were observed with a mutation in either *cact* or the adaptor protein *myd88*. Since both *cact* and *myd88* are thought to be canonical cell autonomous components of the *Tl*→NFκB signaling pathway a plausible explanation is that these genes are not haploinsufficient in the eye assay. In the case of *spz* we cannot exclude the possibility that some other effector molecule mediates the activation of the *Tl*→NFκB pathway.

While our results clearly demonstrate that the *Tl*→NFκB pathway plays a key role in facilitating the degenerative effects of the Aβ42 polypeptide on the eye, the evidence that it also mediates the Aβ42-dependent reduction in life span is less clear cut. The *Tl* allele that is the strongest suppressor in the eye assay, *Tl^r4^*, appears to extend the life span of *UAS-Aβ42*/*elav-GAL4* flies by about a quarter. However, much more modest effects, if any, were observed for two other *Tl* alleles, as well as for mutations in *tub* and *pll*. Several factors could explain why these other mutations didn't greatly extend the life span. For one, it is possible that the mutations we tested do not sufficiently reduce the overall activity of the *Tl*→NFκB signaling pathway as heterozygotes to have much of an impact on the average life span of the *UAS-Aβ42*/*elav-GAL4* flies. Both of the *Tl* alleles are hypomorphs and neither was a strong suppressor in the eye assay. This is also true for the *tub* and *pll* mutations. Given the very strong suppression of the rough eye phenotype observed pGMR- Aβ42 flies that are homozygous for *dl* mutations, it is possible that we would observe a significant suppression of the life span defects in mutants that lack *tub* or *pll* altogether. Another difference is that the life span assay is likely to be a much more indirect measure of the degenerative effects of the Aβ42 polypeptide. While the deleterious effects of the Aβ42 polypeptide on individual cells in each ommatidia can be observed directly, life span depends upon a complex combination of genetic background, environmental conditions and chance circumstances and the neurodegeneration induced by Aβ42 is just one element among many that determine mortality rates. Additionally, there may be specific neuronal circuits whose activity must be maintained above a critical threshold in order to prolong survival. In this case, even if the overall neurodegenerative effects of the Aβ42 polypeptide in the CNS are greatly ameliorated by reducing the activity of the *Tl*→NFκB pathway, this might not be sufficient to substantially increase life span.

It is also possible that reducing the activity of the *Tl*→NFκB signaling pathway by two-fold (in animals heterozygous for a null allele) will not be sufficient in itself to strongly suppress the deleterious effects of the Aβ42 polypeptide on life span. One reason why this might be the case is that neurodegenerative effects of activating the *Tl*→NFκB signaling pathway in the CNS likely depend upon triggering other, cell autonomous pathways or processes after a prolonged or chronic inflammatory response. In this case, it might be necessary to simultaneously alter the activity of one or more of these interacting pathways in addition to *Tl*→NFκB.

Some potential candidates for the participating pathways were uncovered in our genetic screen for genes that modulate the Aβ42 induced rough eye phenotype. For example, we found that a small deficiency, *Df(3)H99,* which removes three pro-apoptotic genes [66], *reaper*, *grim* and *hid*, is a strong suppressor of the rough eye phenotype. This would suggest that the cell death pathway promotes the neurodegenerative effects of the human Aβ42 polypeptide in the *Drosophila* eye. Consistent with an induction of apoptosis, we found that a mutation in *croquemort*, which is required for the phagocytosis of apoptotic cells in *Drosophila* [67], dominantly enhances the rough eye phenotype. A role for apoptosis in the degenerative process would also be consistent with studies on brains of AD patients which have shown that markers for apoptosis are elevated. In this regard it is interesting to note that c-Jun N-terminal kinase (JNK) signaling cascade, which is thought to promote apoptosis, is upregulated in flies by both the *Tl*→NFκB and IMD innate immunity pathways [56]. Moreover, a number of observations support a possible role for the JNK pathway in Aβ42 induced neurodegeneration in the fly as well. First, one of the chromatin modifiers identified in the EP screen was the histone deacetylase Rpd3 (Hdac1)[27]. Rpd3 histone deacetylase activity is required to downregulate the JNK pathway and it functions in complexes with Sin3A and Sap130 [68]. EP insertions that disrupt the function of Rpd3, Sin3A and Sap130 were found to enhance the rough eye phenotype. Second, we found that two mutations in *pucker* (*puc*), which encodes a tyrosine phosphatase that antagonizes the JNK kinase enhance the rough eye phenotype. It has also recently been suggested that activation of the apoptosis cascade, by JNK, promotes the cleavage and hyperphosphorylation of Tau [69]. Tau is a microtubule-associated protein which accumulates in large aggregates in the neurofibrillary tangles of AD patients [70]. The Tau protein can also have neurotoxic effects independent of Aβ42 [70], [71]. The Aβ42 induced rough eye phenotype seems to be connected to the fly *tau* gene as well as since we found that it is enhanced by two independent *tau* mutations. Further studies will be required to elucidate the role of these and other genes in modulating the neuropathological effects of Aβ42 and their connection to the functioning of the *Tl*→NFκB pathway. It will also be important to determine if it is possible to more substantially ameliorate the life span defects of Aβ42 flies by simultaneously manipulating the activity of the *Tl*→NFκB pathway and one of these other pathways.

## Materials and Methods

### Drosophila stocks

All deficiency strains and additional candidate strains were obtained from the Bloomington stock center (http://flystocks.bio.indiana.edu). *GMR-A*β*42* and *UAS-A*β*42* flies are described in [25], *Tl^3^/TM3*, *dl^4^/Cyo, dl^8^/Cyo, hsp83dl/TM3* were gifts from Dr. Govind and Dr. Steward and *Dif^1^* is a gift from Dr. Wu.

### Screen for dominant suppressors and enhancers

*GMR-A*β*42*/Ubx virgin females were used for crosses to males from different deficiencies and candidates strains. Crosses were kept at 22°C, F1 flies were collected and aged for three weeks before being scored for eye phonotype. On average, 50–100 flies that are trans-heterozygote for a deletion or candidate mutant and one copy of *GMR-A*β*42* transgene were scored simultaneously and were compared to their similarly aged siblings who only had one copy of the *GMR-A*β*42* transgene. The suppression/enhancement of the rough eye phenotype was characterized into weak, moderate or strong categories as indicated in the tables.

### Protein analysis

Protein was extracted from fly heads (10–15 heads pooled sample) with sample buffer for Western analysis. Anti-cactus (mouse monoclonal, gift from Dr. Steward, anti-SNF (mouse monoclonal antibody, DSHB, http://dshb.biology.uiowa.edu/) were used. Experimental samples were always compared to control samples of the same age. To account for difference seen among flies with same genotype, we also carried out the Western blot analysis with protein sample extracted from a single fly head.

### Survival Assay

Twenty to 30 young flies were placed in a food vial. Each vial was kept at 29°C. Food vials were changed every 4–5 days, and the dead flies were counted at that time. At least 50 flies were prepared for each genotype, and the experiments were carried out more than three times.
